# Asthma, Allergic Rhinitis, and Atopic Dermatitis Incidence in Korean Adolescents before and after COVID-19

**DOI:** 10.3390/jcm10153446

**Published:** 2021-08-03

**Authors:** Hyo Geun Choi, Il Gyu Kong

**Affiliations:** 1 Department of Otorhinolaryngology-Head & Neck Surgery, Hallym University Sacred Heart Hospital, Anyang 14068, Korea; pupen@naver.com; 2 Hallym Data Science Laboratory, Hallym University College of Medicine, Anyang 14068, Korea; 3 Department of Otorhinolaryngology, Hospital Medicine Center, Seoul National University Hospital, Seoul 03080, Korea

**Keywords:** asthma, allergic rhinitis, atopic dermatitis, incidence, coronavirus, COVID-19, pandemic, cross-sectional studies

## Abstract

With changes in personal habits (masks and handwashing) during the COVID-19 outbreak, the study analyzed the reporting of physician-diagnosed cases (incidence) of allergic diseases (asthma, allergic rhinitis, and atopic dermatitis) using the data for years 2019 and 2020 from the Korean adolescent risk behavior web-based survey (KYRBWS-15 and 16). Altogether, 92,659 adolescents (48,443 in 2019 and 44,216 in 2020) were enrolled. The crude and adjusted odd ratios (ORs) were calculated for each disease in 2020 compared to that in 2019 using multiple logistic regression. Subgroup analyses were performed according to sex and economic status. The incidence of asthma decreased from 1.5% in 2019 to 1.0% 2020 (*p* < 0.001). The incidence of allergic rhinitis in 2019 and 2020 was 19.5% and 16.3%, respectively (*p* < 0.001). Compared with 2019, the adjusted OR (aOR) in 2020 was 0.68 (95% CI = 0.66–0.77, *p* value < 0.001) for asthma and 0.82 (95% CI = 0.78–0.85, *p* < 0.001) for allergic rhinitis. In contrast, there was no statistically significant difference between the incidence of atopic dermatitis in 2019 and that in 2020 (6.4%, vs. 6.4%, *p* > 0.05, respectively). Subgroup analyses results were consistent. In conclusion, there was decrease in the incidence of asthma and allergic rhinitis but not in that of atopic dermatitis from 2019 to 2020.

## 1. Introduction

In 2020, coronavirus disease 2019 (COVID-19) became a global pandemic, and people’s lifestyle has since been dramatically changed to prevent contagious infections. The official encouragement to handwash, mask-wear, and social distance impacts not only the spread of COVID-19 but also that of other infectious diseases and alters the lifestyle habits. In particular, the school-aged children and adolescents had a long period of homeschooling via digital devices, since schools were closed for months during the COVID pandemic under government regulations.

Allergic diseases such as asthma, allergic rhinitis, and atopic dermatitis, which may occur at a relatively young age, impair the patients’ quality of life [1]. Allergic diseases require continuous management and treatment, and their socioeconomic burden has been increasing worldwide [1]. The prevalence of allergic diseases has been dramatically increasing in developing countries [1,2]. In Korea, the increasing trends in asthma, allergic rhinitis, and atopic dermatitis remain controversial [3,4,5,6,7]. However, a recent study with large population data based on the Korean National Health and Nutrition Examination (KNHANES) 2008–2017 reported that the 10-year trend of prevalence of asthma is stable, and those of allergic rhinitis and atopic dermatitis are increasing in school-going children [8].

Efforts to control infectious diseases change many aspects of our daily lives. In South Korea, people have continued to wear a mask, wash hands, and keep social distancing since the first report of cases of COVID-19 in February 2020. As the unprecedented ‘sanitation’ period has been extended by more than one year, it would be worthwhile to evaluate the changes in the occurrence of allergic diseases before and after the introduction of nationwide preventive measures with a clear expectation of the reductions in respiratory infectious diseases and modification of lifestyle habits.

This study was designed to compare the rate of newly diagnosed cases (incidence) of allergic diseases (asthma, allergic rhinitis, atopic dermatitis) in 2019 (before COVID-19) and in 2020 (during the COVID-19 outbreak) in a group of Korean adolescents, based on data from the Korea Youth Risk Behavior Web-based Survey (KYRBWS).

## 2. Materials and Methods

### 2.1. Study Population and Data Collection

This cross-sectional study used data from the Korea Youth Risk Behavior Web-based Survey (KYRBWS), which represented of the national data using statistical methods based on designed sampling and adjusted weighted values. In 2019, the KYRBWS was performed from 3 June through 12 July. In the 2020 KYRBWS, data were collected from 3 August through 13 November. The KYRBWS obtained data from South Korean adolescents using stratified, two-stage (schools and classes) clustered sampling based on data from the education ministry. Sampling was weighted by statisticians, who performed post-stratification analyses and considered the non-response rates and extreme values. Data from the 2019 and 2020 KYRBWS were analyzed. Details of the sampling methods are described on the KYRBWS website [9]. The Centers for Disease Control and Prevention of Korea (KCDC) collected the data, and Korean adolescents from 7th through 12th grade completed the self-administered questionnaire voluntarily and anonymously. The validity and reliability of the KYRBWS have been documented by other studies [10,11].

**Figure 1:**
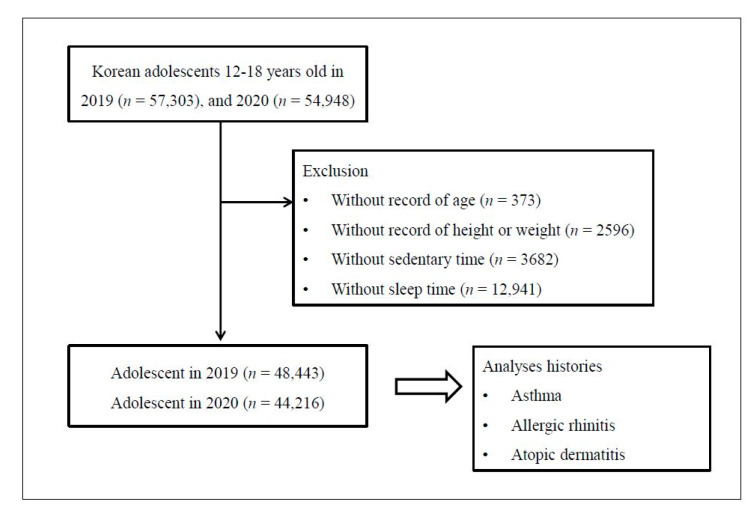
A schematic illustration of participant selection in the present study. Among 112,251 total participants (57,303 in 2019; 54,948 in 2020), 92,659 participants (48,443 in 2019; 44,216 in 2020) aged 12 through 18 years were included.

Of the 112,251 total participants (57,303 in 2019; 54,948 in 2020), the following were excluded from this study: participants without information on age (*n* = 373), height or weight (*n* = 2596), sedentary time (*n* = 3682), and sleep time (*n* = 12,941). Finally, 92,659 participants (48,443 in 2019; 44,216 in 2020) 12 through 18 years old were included in this study (Figure 1). Then, we analyzed the changes in annual cases of asthma, allergic rhinitis, and atopic dermatitis reported by physician between 2019 and 2020.

The ethics committee of Hallym University approved the use of these data. The study was exempted from the need for written informed consent by the Institutional Review Board (2019-09-005). Analyses of data from the KYRBWS were conducted in accordance with the guidelines and regulations provided by the Institutional Review Board of the KCDC. The understanding, reliability, and validity of each question were investigated by the KCDC to verify the applicability of the surveys [9,10]. 

### 2.2. Survey

#### 2.2.1. Exposure

In both 2019 and 2020, the adolescent participants were selected, as stated previously, to represent the entire adolescent population in Korea. In each year’s survey, the new participants were selected independently from the entire Korean adolescent population.

#### 2.2.2. Outcome

Participants were asked their histories of asthma, allergic rhinitis, and atopic dermatitis by the questionnaire including the questions based on the International Study of Asthma and Allergies in Childhood (ISAAC) Phase-Three Manual [12,13]. We investigated the annual rate (incidence) of physician-diagnosed allergic diseases listed as asthma, allergic rhinitis, and atopic dermatitis in the past 12 months according to the response to the question: ‘Have you been diagnosed with asthma/allergic rhinitis/atopic dermatitis by a doctor within last 12 months?’.

#### 2.2.3. Covariate

Body mass index (BMI, kg/m^2^) was calculated using participants’ height and weight. Days of physical activity were measured as the number of days in the past 7 days that the participants had exercised for more than 60 min at intensity high enough to increase their heart rate or respiration. Each of mean sedentary times (hour/day) for study, and leisure were calculated as 5/7 time in weekday plus 2/7 time in weekend. Sleep times were calculated as 5/7 time in weekday plus 2/7 time in weekend. Self-reported economic level was measured on a three-level scale, high/middle/low. Educational levels of father and mother were categorized into three groups:

(1) unknown, missing or below middle school
(2) high school
(3) college, or over

Subjective self-reported health status was categorized into four levels: from very healthy to unhealthy. The subjects’ stress level was surveyed, and the response was categorized as very severe, severe, a little, little, and no stress.

### 2.3. Statistical Analysis

The general characteristics between 2019 and 2020 were compared using linear regression analysis with complex sampling, and chi-square test with Rao–Scott correction, to represent the entire population, as this study was designed to use weighted values. Following the univariate analysis, multiple logistic regression analysis with complex sampling was performed, and odd ratios (ORs) for asthma, allergic rhinitis, and atopic dermatitis diagnosed by the medical doctor in the past 1 year were adjusted for age, BMI, physical exercise, sedentary time for study and leisure, sleep time, sex, economic level, educational level of father and mother, subjective health status, and subjective stress. Crude and adjusted (according to age, BMI, physical exercise, sedentary time for study and leisure, sleep time, sex, economic level, educational level of father and mother, subjective health status, and subjective stress) models were designed. We performed subgroup analysis for sex, because there can be a possibility of sex-specific difference between adolescents in atopic susceptibility [14]. The subgroup analysis for socioeconomic status, which can affect the medical help seeking behavior during the COVID-19 pandemic, was also performed. 

Two-tailed analyses were conducted, and *p*-values lower than 0.05 were considered to indicate significance; 95% confidence intervals (CIs) were also calculated. The weights recommended by the KYRBWS were applied, and thus all results are presented as weighted values. The data were analyzed using SPSS ver. 25.0 (IBM, Armonk, NY, USA).

## 3. Results

### 3.1. General Differences of Variables and Reporting (Incidence) of Allergic Diseases between 2019 and 2020 Korean Adolescent Population

The general characteristics of the included population are presented in Table 1. In 2020, the population was higher in age and BMI (all *p* values < 0.001, Table 1). The physical exercise time, sedentary time for study, and sleep time were decreased (all *p* values < 0.001, Table 1). The sedentary time for leisure was increased in 2020 (4.2 h/day) compared to that in 2019 (3.3 h/day, *p* < 0.001, Table 1). Educational level of father and education level of mother of the participants were higher in 2020 (*p* < 0.001, Table 1). Stress levels were generally lower in 2020 compared to those in 2019, showing that participants with severe level (28.3%) and very severe stress level (11.1%) decreased to 25.7% and 7.5%, respectively (*p* < 0.001, Table 1).

The differences in the reporting (newly diagnosed within last 12 months) of physician-diagnosed allergic diseases between 2019 and 2020 were analyzed. The reporting of asthma was lower in 2020 (1.0% [448/44,216]) than that in 2019 (1.5% [713/48,443], *p* < 0.001, Table 1). The reporting of allergic rhinitis in 2020 was 16.8% [7220/44,216], which was also lower than that of 20.1% reported in 2019 ([9450/48,443], *p* < 0.001, Table 1). However, there was no difference between the reporting of atopic dermatitis in 2019 and 2020 (6.4%, [3121/48,443] vs. 6.4% [2807/44,216], *p* > 0.05, Table 1).

### 3.2. The Adjusted Odd Ratios for Allergic Diseases in 2020 Compared to 2019 in Korean Adolescents

The adjusted OR (aOR) for asthma in 2020 was 0.68 (95% CI = 0.66–0.77, *p* value < 0.001, Table 2). The subgroup analysis for sex and economic level showed that asthma reporting was lower in 2020 than that in 2019. The aOR for asthma was 0.64 (95% CI = 0.54–0.76, *p* < 0.001, Table 2) in men, and 0.73 (95% CI = 0.60–0.89, *p* = 0.002, Table 2) in women. The aOR for asthma was 0.60 (95% CI = 0.50–0.73, *p* < 0.001, Table 2) in high economic level, 0.76 (95% CI = 0.63–0.93, *p* = 0.006, Table 2) in middle economic level, and 0.71 (95% CI = 0.50–1.00, *p* = 0.047, Table 2) in low economic level individuals.

The aOR for allergic rhinitis in 2020 (compared to that in 2019) was 0.82 (95% CI = 0.78–0.85, *p* < 0.001, Table 3). In the subgroup analysis according to sex and economic levels, the result was consistent. Compared to that in 2019, the aOR for allergic rhinitis in 2020 was 0.84 (95% CI = 0.79–0.89, *p* < 0.001, Table 3) in men, 0.79 (95% CI = 0.74–0.83, *p* < 0.001, Table 3) in women, 0.80 (95% CI = 0.76–0.85, *p* < 0.001, Table 3) in the high economic level, 0.82 (95% CI = 0.77–0.87, *p* < 0.001, Table 3) in the middle economic level, and 0.83 (95% CI = 0.75–0.92, *p* < 0.001, Table 3) in the low economic level. 

The aOR for atopic dermatitis in adolescents in 2020 compared to that in 2019 was 0.99 (95% CI = 0.94–1.05, *p* > 0.05, Table 4), which has no statistical significance. The subgroup analysis for the sex and economic levels showed consistent results, with no difference according to sex or economic levels.

## 4. Discussion

We found that among adolescents in Korea, the reporting of asthma and allergic rhinitis in 2020 was significantly lower compared to that in 2019, while the reporting of atopic dermatitis remained constant. To the best of our knowledge, this is the first study that focused on the reporting of allergic diseases in the adolescent population after the onset of COVID-19, correcting for several potential confounding variables. In 2020, considering the fact that hard work has been employed for a holistic blockade of respiratory diseases compared to the previous year, the decrease in newly diagnosed asthma and allergic rhinitis seems highly likely to be related to the direct and indirect changes in lifestyle during the COVID-19 pandemic.

Several studies reported the results that the COVID lockdown or important measures to limit the spread of virus changed lifestyle habits such as diet, physical activity, stress, and sleep adversely [15,16,17]. A decrease in physical activity during the pandemic has been reported in several reports [15,16,18], and it was confirmed that physical activity decreased in the 2020 survey in our data as well. There is a lack of evidence that evaluates the multifaceted changes of COVID-19 on lifestyle-related behaviors in Korea. In our study, the differences were observed in the factors related to the lifestyle before and after the COVID-19 pandemic. In 2020, during the pandemic, the adolescent population had higher BMI, decreased physical activity, and increased sedentary time for leisure, with a more sedentary lifestyle.

Several studies have reported that lack of exercise and excessive sedentary behavior are associated with asthma [19,20,21]. According to a recent report, an increase in the amount of time spent in sedentary activities, such as watching TV, is associated with an asthma attack [22,23]. Lack of exercise and a sedentary lifestyle are associated with obesity. Obesity and allergic diseases are important health problems, and there is evidence that a sedentary lifestyle increases the prevalence of obesity as well as allergies [24]. Obesity is well known as a risk factor for chronic diseases such as cardiovascular disease and type 2 diabetes and is also known as a risk factor for asthma [25,26,27]. There are few studies on the relationship between obesity, lack of exercise, sedentary lifestyle, and rhinitis and dermatitis. One study in adolescents did not report a relationship between obesity and dermatitis [28,29], and no relationship was reported in studies involving obesity and allergic rhinitis [30,31]. A Japanese study of children reported that obesity was associated with asthma but not with allergic rhinitis and dermatitis [32]. The ISAAC study reported an association between obesity and asthma and dermatitis but not allergic rhinitis [33]. 

According to previous studies, the trends of our data related to the lifestyle in 2020 (high BMI, lack of exercise, and increased sedentary time for leisure) are associated with an increased risk of allergies. Nevertheless, the risk of asthma and rhinitis had decreased in the present study in both of crude and final models. Of note, vigorous exercise activity has also been reported as a risk factor for asthma [33]. Exercise-induced asthma is known to occur in 40–90% of asthmatics and in 20% of people without asthma [34,35]. In this regard, as analyzed in our data, a significant decrease in strenuous exercise due to reduced outdoor activity and physical activity during the COVID-19 pandemic may have an influence on the reduction in the diagnosis of asthma. In addition, the effects of other factors that acutely induce allergy symptoms in a short period of time, such as decreased respiratory infection and decreased exposure to inhaled irritants, should be taken into account, as those factors may have contributed to the decreasing allergic diseases, considering that our observation period is relatively short.

We supposed that the decrease in asthma and allergic rhinitis may be linked to the blockade of respiratory infection by COVID-19 preventive measures or that allergy-triggering environmental factors were simultaneously decreased because of reduced socioeconomic activities, leading to a decrease of particle materials or air pollutants. Actually, there are data presenting that the nationwide social distancing and other preventive measures for COVID-19 were associated with the significantly reduced detection rate of enveloped respiratory viruses such as human coronavirus, metapneumovirus, influenza virus, parainfluenza, and respiratory syncytial virus [36,37,38,39,40,41].

Infectious factors have been identified as causative factors of allergic diseases. The respiratory viral infection has been well documented to be related to asthma development and acute exacerbation of asthma. Early childhood exposure to respiratory syncytial virus (RSV) or rhinovirus (RV) is known to be highly associated with the onset of asthma or persistent wheezing later [42]. Furthermore, there is a dose–response relationship between respiratory infection severity in infancy and asthma development in early childhood [43]. A similar relationship has been reported between respiratory viral infection and asthma exacerbation [44,45]. One study reported that lower respiratory tract infection in the first year of life is associated with allergic rhinitis in children [46]. Kansen et al. showed that the concurrent presence of recurrent respiratory tract infections increased the odds of having asthma or allergic rhinitis, while it reduced the odds of having atopic dermatitis [47].

There is a possibility that during the COVID-19 pandemic, wearing the face mask and social distancing may have had a potential impact on decreasing allergic rhinitis symptoms following decrease in the diagnosis of allergic diseases. Usage of the mask since February 2020 may reduce the severity of allergic diseases by lowering the exposure to the inhaled airborne particles with the physical filtration of allergens or air pollutants and humidification of the breathing [48]. Coincidence of pollen season and the COVID-19 pandemic led people to spend less time outdoors and may have resulted in the reduction in allergy symptoms [49].

There are several limitations associated with this study. Although we suspected that strict personal quarantine practices implemented with the COVID-19 pandemic would have contributed to reducing the overall infectious disease, we did not present objective data on whether the incidence of specific infectious diseases decreased during the period of the actual allergic disease diagnosis. Since we investigated the subjects with allergic diseases diagnosed within the past 1 year of the survey, it is highly likely that acute exacerbation of patients previously diagnosed with allergic diseases one year prior to the survey was also included in addition to the subjects with the first diagnosis of allergic diseases. Thus, the incidence analyzed in this study might include the first-diagnosed allergic disease and the acute exacerbations of the previously diagnosed allergic disease. We did not provide other important data for lifestyle changes after the COVID-19 pandemic such as dietary habits and nutrition. In fact, studies from some European countries have reported that changes in eating habits have occurred during the COVID-19 pandemic, primarily due to a significant increase in the consumption of snacks and sweets [50,51]. It has been reported the children with respiratory allergies have incorrect eating habits such as snacking between meals and eating before bedtime [52]. It cannot be excluded that the diagnosis of allergic disease may have decreased through reduction in hospital use owing to a reluctance to visit healthcare settings or the limitation of medical resources as reported after the COVID-19 pandemic [53,54,55]. Due to the relatively low mortality or morbidity of allergic rhinitis, during the COVID-19 pandemic, patients with allergic rhinitis might have minimized their hospital visits to avoid the risk of respiratory infection. In addition, in the case of allergic rhinitis, the relatively high accessibility of OTC drug use compared to that in asthma raises the possibility of a decrease in hospital visits during the pandemic. However, in the same period, the incidence of atopic dermatitis has not changed, and the incidence of asthma with relatively high morbidity and mortality has reduced more than that of allergic rhinitis. The differences in the trend in each allergic disease might explain that there was a limited effect of the decrease of hospital use on the reduction of the incidence of asthma and allergic rhinitis. Despite these limitations, we would emphasize that the results of this study are important as objective evidence for an ambiguous, but interesting, relationship between allergic diseases and the drastic changes in our daily life after the COVID-19 pandemic. 

## 5. Conclusions

Reports of asthma and allergic rhinitis decreased during the COVID-19 pandemic, while there was no change in reports of atopic dermatitis. Reports of allergic diseases may have decreased since the onset of the COVID-19 pandemic with infection preventive measures. Further investigations are needed on the association between the decrease in the incidence of allergic disease and in infection or lifestyle changes owing to the COVID-19 infection prevention measures.

## Figures and Tables

**Table 1:** General characteristics of participants.

General Characteristics	Participated Year		
	2019	2020	*p*-Value
Total number, *n* (% *)	48,443 (100.0)	44,216 (100.0)
Age (years, mean * (SD))	15.0 (1.7)	15.1 (1.7)	<0.001 ^†^
BMI (kg/m^2^, mean * (SD))	21.4 (3.5)	21.6 (3.7)	<0.001 ^†^
Physical exercise (days/week, mean * (SD))	2.0 (2.1)	1.9 (2.1)	<0.001 ^†^
Sedentary time for study (hour/day, mean * (SD))	6.6 (3.7)	6.0 (3.3)	<0.001 ^†^
Sedentary time for leisure (hour/day, mean * (SD))	3.3 (2.2)	4.2 (2.7)	<0.001 ^†^
Sleep time (hour/day, mean * (SD))	7.0 (1.5)	6.9 (1.5)	<0.001 ^†^
Sex, *n* (% *)			0.498
Male	24,917 (51.3)	23,103 (52.5)
Female	23,526 (48.7)	21,113 (47.5)
Economic level, *n* (% *)			0.322
High	18,992 (39.6)	17,381 (40.3)
Middle	23,376 (48.1)	21,228 (47.5)
Low	6075 (12.3)	5607 (12.2)
Educational level of father, *n* (% *)			<0.001 ^‡^
Unknown, missing, below middle school	23,775 (48.3)	17,957 (39.7)
High school	7385 (14.8)	7519 (16.2)
College or over	17,283 (36.8)	18,740 (44.0)
Educational level of mother, *n* (% *)			<0.001 ^‡^
Unknown, missing, below middle school	23,050 (47.0)	16,925 (37.5)
High school	8637 (17.7)	8941 (19.6)
College or over	16,756 (35.3)	18,350 (42.9)
Subjective health status, *n* (% *)			0.071
Very healthy	12,911 (26.4)	12,283 (27.3)
Healthy	21,444 (44.2)	19,131 (43.4)
Normal	10,749 (22.4)	9742 (22.2)
Unhealthy	3339 (70.)	3060 (7.1)
Stress level, *n* (% *)			<0.001 ^‡^
No stress	1805 (3.6)	1597 (3.5)
Little	7579 (15.4)	8128 (18.2)
A little	20,090 (41.6)	19,907 (45.2)
Severe	13,605 (28.3)	11,231 (25.7)
Very severe	5364 (11.1)	3353 (7.5)
Asthma, *n* (% *)	713 (1.5)	448 (1.0)	<0.001 ^‡^
Allergic rhinitis, *n* (% *)	9450 (20.1)	7220 (16.8)	<0.001 ^‡^
Atopic dermatitis, *n* (% *)	3121 (6.4)	2807 (6.4)	0.739

* Estimated mean or rate-adjusted recommended weighted value; ^†^ linear regression analysis with complex sampling, significance at *p* < 0.05; ^‡^ chi-square test with Rao–Scott correction, significance at *p* < 0.05.

**Table 2:** Odd ratios of asthma in 2020 compared to those in 2019 in total participants and subgroup.

Asthma	Event/Total (*n*, %)		OR (95% CI)			
	2019	2020	Crude	*p*-Value	Adjusted ^†^	*p*-Value
Total participants (*n* = 92,659)	713/48,443 (1.5)	448/44,216 (1.0)	0.69 (0.61–0.78)	<0.001 *	0.68 (0.60–0.77)	<0.001 *
Sex						
Men (*n* = 48,020)	413/24,917 (1.7)	263/23,103 (1.1)	0.67 (0.57–0.79)	<0.001 *	0.64 (0.54–0.76)	<0.001 *
Women (*n* = 44,639)	300/23,526 (1.3)	185/21,113 (0.9)	0.70 (0.58–0.85)	<0.001 *	0.73 (0.60–0.89)	0.002 *
Economic level						
High (*n* = 36,373)	305/18,992 (1.6)	186/17,381 (1.1)	0.63 (0.52–0.76)	<0.001 *	0.60 (0.50–0.73)	<0.001 *
Middle (*n* = 44,604)	312/23,376 (1.3)	203/21,228 (1.0)	0.74 (0.62–0.89)	0.001 *	0.76 (0.63–0.93)	0.006 *
Low (*n* = 11,682)	96/6075 (1.6)	59/5607 (1.1)	0.70 (0.50–0.98)	0.036 *	0.71 (0.50–1.00)	0.047 *

* Multiple logistic regression analysis with complex sampling, significance at *p* < 0.05; ^†^ Adjusted for age, BMI, physical exercise, sedentary time for study and leisure, sleep time, sex, economic level, educational level of father and mother, subjective health status, and subjective stress.

**Table 3:** Odd ratios in allergic rhinitis of 2020 compared to those in 2019 in total participants and subgroup.

Allergic Rhinitis	Event/Total (*n*, %)		OR (95% CI)			
	2019	2020	Crude	*p*-Value	Adjusted ^†^	*p*-Value
Total participants (*n* = 92,659)	9450/48,443 (19.5)	7220/44,216 (16.3)	0.80 (0.77–0.84)	<0.001 *	0.82 (0.78–0.85)	<0.001 *
Sex						
Men (*n* = 48,020)	4554/24,917 (18.3)	3611/23,103 (15.6)	0.84 (0.79–0.89)	<0.001 *	0.84 (0.79–0.89)	<0.001 *
Women (*n* = 44,639)	4896/23,526 (20.8)	3609/21,113 (17.1)	0.77 (0.73–0.82)	<0.001 *	0.79 (0.74–0.83)	<0.001 *
Economic level						
High (*n* = 36,373)	3985/18,992 (21.0)	3038/17,381 (17.5)	0.80 (0.75–0.84)	<0.001 *	0.80 (0.76–0.85)	<0.001 *
Middle (*n* = 44,604)	4280/23,376 (18.3)	3249/21,228 (15.3)	0.81 (0.77–0.86)	<0.001 *	0.82 (0.77–0.87)	<0.001 *
Low (*n* = 11,682)	1185/6075 (19.5)	933/5607 (16.6)	0.80 (0.73–0.89)	<0.001 *	0.83 (0.75–0.92)	<0.001 *

* Multiple logistic regression analysis with complex sampling, significance at *p* < 0.05; ^†^ adjusted for age, BMI, physical exercise, sedentary time for study and leisure, sleep time, sex, economic level, educational level of father and mother, subjective health status, and subjective stress.

**Table 4:** Odd ratios in atopic dermatitis of 2020 compared to those in 2019 in total participants and subgroup.

Atopic Dermatitis	Event/Total (*n*, %)		OR (95% CI)			
	2019	2020	Crude	*p*-Value *	Adjusted ^†^	*p*-Value *
Total participants (*n* = 92,659)	3121/48,443 (6.4)	2870/44,216 (6.3)	0.94 (0.94–1.05)	0.739	0.99 (0.94–1.05)	0.750
Sex						
Men (*n* = 48,020)	1364/24,917 (5.5)	1246/23,103 (5.4)	0.99 (0.92–1.08)	0.852	0.98 (0.90–1.06)	0.546
Women (*n* = 44,639)	1757/23,526 (7.5)	1561/21,113 (7.4)	1.00 (0.93–1.07)	0.924	1.00 (0.93–1.08)	0.939
Economic level						
High (*n* = 36,373)	1205/18,992 (6.3)	1099/17,381 (6.3)	0.99 (0.91–1.07)	0.776	1.00 (0.92–1.08)	0.966
Middle (*n* = 44,604)	1458/23,376 (6.2)	1293/21,228 (6.1)	0.98 (0.91–1.06)	0.648	0.98 (0.90–1.06)	0.976
Low (*n* = 11,682)	458/6075 (7.5)	415/5607 (7.4)	1.03 (0.89–1.19)	0.722	1.02 (0.88–1.17)	0.829

* Multiple logistic regression analysis with complex sampling, significance at *p* < 0.05; ^†^ adjusted for age, BMI, physical exercise, sedentary time for study and leisure, sleep time, sex, economic level, educational level of father and mother, subjective health status, and subjective stress.

## Data Availability

All data are available upon reasonable request from the corresponding author.
